# Mycorrhizal Response to Experimental pH and P Manipulation in Acidic Hardwood Forests

**DOI:** 10.1371/journal.pone.0048946

**Published:** 2012-11-08

**Authors:** Laurel A. Kluber, Sarah R. Carrino-Kyker, Kaitlin P. Coyle, Jared L. DeForest, Charlotte R. Hewins, Alanna N. Shaw, Kurt A. Smemo, David J. Burke

**Affiliations:** 1 Department of Biology, Case Western Reserve University, Cleveland, Ohio, United States of America; 2 The Holden Arboretum, Kirtland, Ohio, United States of America; 3 Department of Environmental and Plant Biology, Ohio University, Athens, Ohio, United States of America; 4 Department of Biological Sciences, Kent State University, Kent, Ohio, United States of America; DOE Pacific Northwest National Laboratory, United States of America

## Abstract

Many temperate forests of the Northeastern United States and Europe have received significant anthropogenic acid and nitrogen (N) deposition over the last century. Although temperate hardwood forests are generally thought to be N-limited, anthropogenic deposition increases the possibility of phosphorus (P) limiting productivity in these forest ecosystems. Moreover, inorganic P availability is largely controlled by soil pH and biogeochemical theory suggests that forests with acidic soils (i.e., <pH 5) are particularly vulnerable to P limitation. Results from previous studies in these systems are mixed with evidence both for and against P limitation. We hypothesized that shifts in mycorrhizal colonization and community structure help temperate forest ecosystems overcome an underlying P limitation by accessing mineral and organic P sources that are otherwise unavailable for direct plant uptake. We examined arbuscular mycorrhizal (AM) and ectomycorrhizal (EcM) communities and soil microbial activity in an ecosystem-level experiment where soil pH and P availability were manipulated in mixed deciduous forests across eastern Ohio, USA. One year after treatment initiation, AM root biomass was positively correlated with the most available P pool, resin P, while AM colonization was negatively correlated. In total, 15,876 EcM root tips were identified and assigned to 26 genera and 219 operational taxonomic units (97% similarity). Ectomycorrhizal richness and root tip abundance were negatively correlated with the moderately available P pools, while the relative percent of tips colonized by Ascomycetes was positively correlated with soil pH. Canonical correspondence analysis revealed regional, but not treatment, differences in AM communities, while EcM communities had both treatment and regional differences. Our findings highlight the complex interactions between mycorrhizae and the soil environment and further underscore the fact that mycorrhizal communities do not merely reflect the host plant community.

## Introduction

Although temperate forests are assumed to be nitrogen (N)-limited [1], current N deposition and possible ecosystem N saturation trends suggest that forests could become phosphorus (P)-limited [2], [3], [4], [5]. This P limitation scenario is exacerbated in those forests with acidic soils or receiving significant anthropogenic acid deposition, such as mixed deciduous forests of the northeastern United States [6]. When soil pH is below 5.5, Al^3+^ becomes mobilized and binds to inorganic P, rendering it less available for plant uptake. Despite a biogeochemical expectation of P limitation, evidence for P limitation in hardwood forests has been lacking [4], [7], suggesting that ecosystems can compensate for reduced mineral P availability. However, several recent studies have found evidence for P limitation in some acidic hardwood forests [8], [9] and have alluded to possible compensation mechanisms.

Although plants can utilize a variety of mechanisms to increase P uptake, the most efficient and widely utilized method is mycorrhizal colonization [10]. Mycorrhizal fungi form a symbiotic relationship with plant roots where fungi provide plants with increased nutrient uptake in exchange for carbon (C) derived from photosynthesis. Mixed deciduous forests contain trees that form both arbuscular mycorrhizal (AM) and ectomycorrhizal (EcM) associations and there is ample evidence that these mycorrhizae increase P uptake and nutrition in plants, although the P uptake rate and efficiency can vary among mycorrhizal species [5], [11], [12], [13]. Both AM and EcM fungi have the ability to produce extracellular enzymes to hydrolyze organic P; however, EcM have a much greater capacity to do so [11]. Furthermore, some EcM are known to produce organic acids as a means to acquire P through mineral weathering [14], [15], [16]. Although AM fungi have not been shown to produce organic acids to enhance P uptake [17], they are known to alleviate stress on plants growing in acidic soils [18] and immobilize Al^3+^ [19], potentially improving host plant P status.

Most previous studies examining the effects of soil pH on EcM fungi suggest that acidic conditions reduce EcM colonization and alter community structure [20], , although some have seen no influence of acidification on community structure [23]. A number of liming experiments also reported EcM community shifts with increased soil pH [24], [25], [26]. For example, Wallander et al. [25] increased pH from 4.5 to 5.8 and suggested that the EcM community shifted from one specialized for organic N acquisition to one that utilizes inorganic N, although P availability was not addressed. Likewise, Lilleskov et al. [27] observed that as soil N availability increased, EcM communities shifted towards taxa possibly specialized for nutrient uptake under acidic, P-limiting conditions. However, these studies did not measure or directly address P availability.

A number of studies have examined the response of mycorrhizal colonization to changes in P availability and a meta-analysis by Treseder [28] determined that P fertilization led to a moderate decline in mycorrhizal abundance, averaging 32% across studies. However, it is important to note that the majority of these studies focused on AM fungi and all were conducted in grasslands, agricultural settings, or tropical forests. Additional work has demonstrated that AM colonization and community structure can vary with soil pH [29], [30], and Dumbrell et al. [31] determined that soil pH had a greater influence on AM fungal communities than host plant species. Phosphorus availability has also been shown to influence EcM community structure [32], [33], and it has been suggested that overall EcM diversity may be more important for P uptake than the EcM community composition [34]. However, the breadth of studies addressing mycorrhizal communities and P biogeochemistry in temperate forests is narrow.

We contend that a clear understanding of how soil pH and P availability influence both AM and EcM fungi is lacking, partly because this relationship has been understudied in temperate forests where P limitation is likely under certain soil conditions [9]. We used molecular-based techniques to quantify AM and EcM community changes and extracellular enzyme assays to estimate soil microbial activity in an ecosystem-level manipulative experiment where pH and inorganic P availability were elevated in acidic hardwood forests. Current soil microbial communities likely reflect a response to chronic ecosystem acidification [6], [9], and alleviating pH and nutrient stress may shift communities toward pre-acidification conditions as trees become less dependent on mycorrhizae for P uptake or other mycorrhizal taxa are favored under those conditions. We therefore hypothesized that in response to elevated pH and P: 1) mycorrhizal colonization would increase with elevated pH and decrease in response to elevated P; 2) altered mycorrhizal communities would result from changing soil conditions; and 3) the activity of extracellular enzymes responsible for organic P acquisition would decrease, thus indicating reduced reliance on organic P sources.

## Methods

### Site Description

We initiated our experimental treatments in six mixed deciduous forests in eastern Ohio, USA. The forests are dominated by oak (*Quercus* spp.), maple (*Acer* spp.), and beech (*Fagus grandifolia*), and are evenly divided between two physiographic regions: previously glaciated sites in northern Ohio, and unglaciated sites in southern Ohio. Regions with differing ages and developmental processes were utilized to examine large-scale trends in how forests respond to experimental pH and P manipulation. Forests in the glaciated region are located on silty loam Hapludalfs or loamy Endoaqualfs, and forests from the unglaciated region are on loamy Hapludalfs, silty loam Dystrudepts, and silty loam Hapludults. The average soil pH is 4.34 in the glaciated region and 4.68 in the unglaciated region. The glaciated region has an average temperature of 8.1°C and receives an average of 120 cm precipitation. The unglaciated region has an average temperature of 10.7°C and receives an average of 100 cm precipitation.

In August 2009, 72 800 m^2^ (20×40 m) plots were established and treatments were applied in a randomized complete block design with two regions (glaciated and unglaciated), six forests (i.e., blocks) and four treatments (control, elevated pH, elevated P, and elevated pH+P). Hi-Ca lime (The Andersons, Maumee, OH, USA) was added to the elevated pH plots to attain a target pH of 5.8 to 6.2 for the top 7 cm; this range has been shown to immobilize reactive Al in these soils [35]. On average, the glaciated and unglaciated sites were amended with 11.4 and 7.3 Mg ha^−1^ Hi-Ca lime, respectively. Elevated P plots were amended with 41.8 kg P ha^−1^ using triple super phosphate (TSP; The Andersons, Maumee, OH, USA). The elevated pH+P plots were amended with both Hi-Ca lime and TSP. Both the Hi-Ca lime and TSP were applied using hand operated spreaders. A detailed description of the study location, soil properties and experimental design can be found in DeForest et al. [9].

### Sample Collection

Soil collection began 23 Aug 2010 (approximately two months after DeForest et al. [9]) with one block from each forest sampled each week, alternating between glaciated and unglaciated forests, for a total of 12 plots per week for six weeks. This staggered sampling was necessary to allow for the timely morphotyping of EcM root tips. The weather remained consistent for the duration of the sampling effort and no effect of sampling date was detected.

Mineral soil cores (4 cm diameter, 5 cm depth) were taken 1 m from the base of randomly selected trees greater than 6 cm in diameter at breast height, after the O horizon (<2 cm) was removed. Within each plot, 10 soil cores were combined to create a representative composite sample for each plot. Soils were transported on ice and gently sieved (2 mm) to separate the roots and homogenize the soil. Roots and soils were stored at 4°C until processing.

### Soil and Enzyme Analysis

Gravimetric water content was determined by drying a subsample from each plot for 48 h at 105°C. Soil pH was measured after 10 g field moist soil was added to 20 ml deionized water and shaken for 30 min. Total C and N were determined by combustion using an ECS 4010 CHNSO elemental analyzer (Costech Analytical, Valencia, CA). Phosphorus was sequentially fractioned using previously described methods [9]. The most labile fraction, resin P (i.e., PO_4_^3−^), was extracted using anion exchange membranes (AEM; GE Infrastructure: Water & Process Technologies, Watertown, MA) immediately after sieving. Soil P was further fractionated by extracting with 0.5 M NaHCO_3_ and 0.1 M NaOH to yield the bicarb P and hydroxide P fractions, respectively.

Potential extracellular enzyme activity (EEA) associated with C, N, and P cycling were measured on fresh soils within 24 h of collection. Soil slurries were prepared by homogenizing 5 g field moist soil in 500 ml 50 mM acetate buffer (pH = 5). Two C-acquiring enzymes (β-1,4-glucosidase and cellobiohydrolase), two N-acquiring enzymes (β-N-acetylglucosaminidase and leucine aminopeptidase), and two P-acquiring enzymes (phosphomonoesterase and phosphodiesterase) were measured in black 96-well plates using fluorogenic methylumbelliferone (MUF)-linked substrates as previously described [9], [36]. All EEAs are expressed per gram of dry soil.

### Root Sorting and Processing

The composited roots samples from each plot were washed with deionized water to remove adhered soil, examined under a three diopter lighted magnifier (Waldmann Lighting Group, Wheeling, IL), and sorted into three categories: AM roots (fresh, non-woody roots from AM trees), EcM roots (roots with EcM root tips), and woody roots (no AM or EcM colonization, all diameter sizes included). Because we were focused on the response of mycorrhizae associated with trees, herbaceous roots were discarded. Each category was then verified at 10–40×magnification under a stereomicroscope. Ectomycorrhizal root tips from each plot were removed from the root system and sorted into morphotypes based on size, shape, color, texture, branching pattern, and presence of rhizomorphs or hyphae [37]. Roots from which the EcM tips were removed were added to the woody root category. The number of root tips per morphotype was recorded. Woody roots were dried at 65°C for 90 h. AM roots were surface sterilized with 70% ethyl alcohol and lyophilized (freeze-dried) in a Genesis 25 EL (VirTis, Gardiner, NY). Morphotyped EcM root tips and lyophilized AM roots were stored at −70°C prior to molecular analysis. All fresh root samples were processed within one week of sampling.

### Molecular Identification of AM Communities and Colonization

Lyophilized AM roots were ground in liquid nitrogen with a mortar and pestle prior to DNA extraction. Genomic DNA was isolated from the lyophilized and ground root tissue with a bead beating and phenol/chloroform protocol [38]. A 550 bp region of the AM fungal 18S rRNA gene was PCR-amplified with primers AM1 [39] and NS31 [40]. Each 50 µl reaction contained 2 units of GoTaq® DNA Polymerase (Promega Corp., Madison, WI), 1X GoTaq Flexi® buffer, 2 mM MgCl_2_, 0.8 mM dNTP mix, 0.5 µg/µl bovine serum albumin, 0.2 µM of each primer, and 1 µl of undiluted template DNA. Thermal cycling conditions were 4 min at 94°C, followed by 32 cycles of 94°C for 30 s, 58°C for 1 min, and 72°C for 90 s, with a final extension of 72°C for 5 min.

Terminal restriction fragment length polymorphism (TRFLP) profiling was used to examine the structure of the AM root communities. Amplicons were generated with the NS31 primer fluorescently labeled with HEX using the PCR conditions described above and cut with the endonuclease *Hinf*I (Promega). The *Hinf*I enzyme has been previously shown to generate large numbers of distinct AM terminal restriction fragments (TRFs) [38]. Although separation between some TRFs in the genera *Glomus* and *Acaulospora* was difficult when using *Hinf*I in TRFLP [38], DNA sequences from our samples returned only *Glomus* spp. (see Results), indicating that *Hinf*I is appropriate for TRFLP in our root samples. TRFLP profiles were generated at the Cornell University Life Sciences Core Laboratories Center (Ithaca, NY) using an Applied BioSystems 3730xl DNA Analyzer with the GS600 LIZ size standard. GeneMapper v4.0 software (Applied Biosystems, Foster City, CA) was used to analyze and bin TRF peaks following methods described by Avis and Feldheim [41].

Although TRFLP is a proven method for detecting overall community trends, it provides no taxonomic information on the community inhabitants. To identify some of the AM taxa encountered in this study we constructed a single clone library. The clone library was constructed by pooling 10 µl of DNA extracts from each plot within treatment types, resulting in a single sample for each treatment (i.e., four samples total, one each for control, elevated pH, elevated P, and elevated pH+P). The pooled DNA samples were PCR-amplified using unlabeled primers AM1 and NS31 using the conditions described above. The resulting PCR products were gel-extracted using the Wizard® SV Gel and PCR Clean-Up System (Promega) following the manufacturer’s protocol. Equal volumes of each cleaned gel extract were pooled and quantified using gel electrophoresis. The ligation and transformation reaction was performed with the pGEM®-T Easy Vector System (Promega) according to the manufacturer’s protocol using JM109 competent cells (Promega). Plasmids were purified with the Wizard® Plus SV Minipreps DNA Purification System (Promega) and inserts were amplified with SP6 and T7 plasmid primers for use in sequencing. The SP6/T7 PCR reactions were essentially as described above except the final volume was 25 µl and 1 unit of DNA polymerase was used. Thermal cycling conditions were 10 min at 96°C, followed by 35 cycles of 96°C for 15 s, 40°C for 30 s, and 72°C for 80 s, with a final extension of 72°C for 7 min. The amplified inserts were cleaned using the Wizard® SV Gel and PCR Clean-Up System (Promega) according to the manufacturer’s protocol and directly sequenced with plasmid primer SP6 using the BigDye® Terminator v3.1 Cycle Sequencing Kit (Applied Biosystems, Foster City, CA). Sequencing was conducted on an Applied BioSystems 3730xl capillary DNA sequencer at the Life Sciences Core Laboratories Center (Cornell University). The sequences were checked for quality, trimmed, and grouped into Operational Taxonomic Units (OTUs) of 97% sequence similarity [42], [43] using Geneious Pro [44]. Sequence identities were determined by comparison to the GenBank database (http://www.ncbi.nlm.nih.gov/genbank/) using the BLAST algorithm. In silico digests of sequences from the clone library were performed in Geneious Pro using the *Hinf*I restriction site. Sequences from the AM clone library have been deposited in the EMBL/GenBank/DDJB databases under accession numbers JQ654497–JQ654585.

The copy number of AM 18S genes was assessed with quantitative PCR (qPCR), which was used as an estimate of AM root colonization in the freeze-dried root samples. Quantitative PCR of AM rDNA has been shown to be a reliable technique for studying AM colonization in roots [45], [46]. In addition, quality control guidelines established by Bustin et al. [47] were followed to ensure robust results. Three replicate qPCR reactions were conducted for each sample and run on a MiniOpticon™ real-time PCR detection system (Bio-Rad). The 25 µl qPCR reactions contained 1 µl of template DNA, 1X SsoAdvanced™ SYBR® Green Supermix (Bio-Rad), and 0.2 µM of each primer. The primers used were NS31 and AM1 (described above). Our sequencing effort (see Results) indicated that these primers were specific to AM fungi in our root samples, as only AM fungi were detected. The thermal cycling conditions were 95°C for 4 min followed by 30 cycles of 95°C for 30 s, 58°C for 1 min, and 72°C for 90 s with plate reads after every 72°C step. The specificity of the qPCR reactions was determined by melt curves (60°C−95°C) and by running a subset of the reactions on a 2% agarose gel.

The gene copy number in the root samples was determined by comparing the quantification cycle (C_q_) in the sample reactions to a standard curve using the CFX Manager™ software, version 2.0 (Bio-Rad). Eight qPCR runs were conducted, each with their own standard curve. Standard curves were generated using a transformed plasmid containing an AM 18S sequence from our sequencing effort that was quantified with the Quant-it™ PicoGreen® dsDNA Reagent (Invitrogen). Each run had a four point standard curve that ranged in value from 10^6^ to 10^3^ copies. The C_q_ was determined manually for each run, such that the reaction efficiency and r^2^ of the standard curve were optimized. The r^2^ for the standard curves ranged from 0.991–0.998 and the efficiencies of the runs ranged from 97.17%–100.23%. All sample reactions fell within this standard curve and all no template controls (NTCs) were below detection. The C_q_ standard deviation between the three sample replicates was low; therefore no sample replicates were removed from the analysis.

### Molecular Identification of EcM Root Tips

Ectomycorrhizal root tips from each morphotype were crushed with a sterile pestle and DNA was extracted using the Extract-N-Amp™ Plant PCR Kit (Sigma-Aldrich, St. Louis, MO). Manufacturer’s instructions were followed, except that only 20 µl of Extract Solution and Dilution Solution were used. Depending on the size of the EcM tips, one or two tips per morphotype were used per DNA extraction to yield sufficient DNA for PCR amplification. The fungal internal transcribed spacer (ITS) region was amplified with the ITS1F [48] and ITS4 [49] primers, the PCR reaction mix supplied with the Extract-N-Amp kit, and previously described thermal cycling conditions [50]. Products from the PCR were checked on a 2% agarose gel. In the event of an unsuccessful PCR, the reaction was repeated. If the reaction failed twice, additional root tips from the morphotype were extracted using a modified CTAB and bead-beating protocol [51] and were again subjected to PCR amplification. PCR products yielding a single band were submitted to the Genome Sequencing and Analysis Core Facility at Duke University (Durham, NC) for robotic cleanup and direct Sanger sequencing using an Applied BioSystems 3730xl DNA analyzer. All samples were initially sequenced from the ITS1F primer. However, samples yielding poor sequences were subsequently sequenced from the ITS4 primer. Morphotypes were removed from the dataset if they could not be amplified, did not yield a single PCR band, or did not yield a readable sequence after two sequencing attempts. Fungal ITS sequences were checked for quality and trimmed using Geneious Pro [44] and identified using BLAST searches to the GenBank (http://www.ncbi.nlm.nih.gov/Genbank/) and Unite (http://unite.ut.ee/) databases. All root tips within a given morphotype were assigned to the identified taxa for statistical analysis. Morphotypes with clearly non-EcM sequences were discarded from further analysis. Additionally, OTUs of 97% sequence similarity were determined using Geneious Pro [44] to approximate species diversity [42], [43]. Ectomycorrhizal sequences have been deposited in the EMBL/GenBank/DDJB databases under the accession numbers HE820312−HE820693.

### Statistical Analysis

A linear mixed effects (LME) model was used to test the effect of treatment on soil chemistry, EEA, AM and woody root biomass, and mycorrhizal colonization using the R ‘nlme’ package version 2.13.0 [52] as previously described by DeForest et al. [9] with region and treatment as fixed effects and forests as the random effect. Spearman rank order correlation analysis using SigmaStat v3.5 (Systat Software Inc., CA) was performed to evaluate the overall relationship between soil pH and P pools, and root biomass and mycorrhizal colonization.

Canonical correspondence analysis (CCA) was performed to test for the effect of region, forest, treatment, pH, and P availability on the composition of the mycorrhizal communities. Although CCA ordination generally yields lower variance explained compared to other ordination methods [53], this method was chosen because we wanted to focus on how community structure is influenced by the treatments and region. Peak area data from the AM TRFLP profiles were normalized by total fluorescence of the individual sample (i.e., plot). Ectomycorrhizal community analysis was performed on the number of root tips per genus in each plot. Rare peaks and genera (found in only one plot) were removed from the data set, as were plots with no EcM root tips. The AM and EcM community matrices were log transformed and CCA analysis was performed individually using PC-ORD v5 (MjM Software Design, Gleneden Beach, OR). CCA was performed using the Hill’s scaling method and 1000 Monte Carlo permutations to test for ordination significance and the relationship between the communities and experimental parameters. Minitab v16 (Minitab Inc., State College, PA) was used to perform multivariate analysis of variance (MANOVA) on CCA ordination scores to test for significant clustering by region or treatment. This method was chosen because it is able to account for the more complex study design [53] and has been shown to be a robust method for detecting treatment effects in community ordinations [54], [55]. Rarefaction curves were constructed using Estimate S [56] to relate the number of EcM OTUs to the number of root tips.

## Results

### Soil Chemistry and Enzyme Activity

The effect of region and treatment on soil pH and P is presented in Table 1. Soil pH did not differ by region, and was significantly increased in the elevated pH and elevated pH+P plots. Phosphorus additions in the elevated P and elevated pH+P plots led to a significant increase in the resin P pool, although bicarb P and hydroxide P pools were not significantly different from controls. However, the glaciated soils had significantly more (i.e., 2-fold) P in these pools than the unglaciated soils. There were no significant effects of treatment on total N or total C, although both were significantly greater (i.e., 2-fold) in glaciated soils (*P*<0.05; Table S1). All EEAs were significantly greater in the glaciated soils (*P*<0.05), although phosphodiesterase was the only EEA to have a significant treatment effect (*P* = 0.04; Table S1). All treatments had lower mean phosphodiesterase activity compared to controls; however, no individual comparisons were significant using the LME model (Table S1).

**Table 1:** The effect of region and treatments on soil pH and phosphorus with mean values and standard errors.

Variable	*P*-value			Glaciated				Unglaciated			
	Region	Trt	Region x Trt	Control	Elevated pH	ElevatedP	Elevated pH+P	Control	Elevated pH	ElevatedP	Elevated pH+P
Soil pH	0.34	<0.01	0.07	4.43 (0.04)	5.89** (0.03)	4.51 (0.03)	5.89** (0.03)	4.88 (0.05)	6.03** (0.07)	5.03 (0.07)	5.85**(0.07)
Resin P(mg P kg^−1^)	0.64	<0.01	0.01	0.86 (0.04)	0.28** (0.02)	2.39** (0.16)	1.25 (0.07)	0.66 (0.03)	0.39 (0.03)	3.38** (0.10)	1.40** (0.14)
Bicarb P(mg P kg^−1^)	0.02	0.72	0.48	22.44 (0.85)	20.07 (1.35)	17.38 (0.34)	21.99 (1.57)	12.13 (0.38)	9.70 (0.31)	13.87 (0.53)	12.79 (0.27)
Hydroxide P(mg P kg^−1^)	0.02	0.49	0.66	48.64 (2.30)	43.95 (1.59)	51.41 (2.91)	48.96 (1.83)	20.19 (0.59)	23.42 (0.71)	26.79 (1.09)	22.61 (0.62)

*P*-values for the effect of region and treatments from the LME model with forest blocks as the random effect (n = 9). Asterisks denote a significant difference, in comparison to controls, at *P*<0.05 (**) and *P*<0.10 (*).

### Mycorrhizal Fungi and Root Biomass

**Figure 1:**
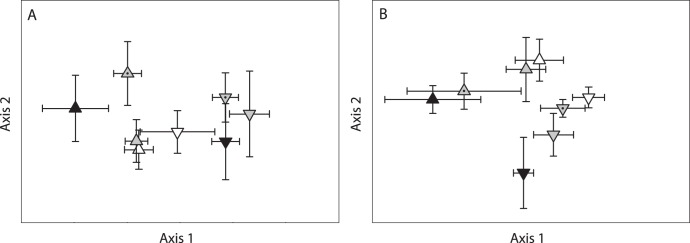
CCA ordinations showing the effect of location, treatment, pH, and P availability on the on AM (A) and EcM (B) communities. Region is denoted by shape: glaciated (triangles) and unglaciated (inverted triangles) and treatment is denoted with color: control (white), elevated pH (grey), elevated P (dotted grey), and elevated pH+P (black). Centroids and error bars represent the mean and standard errors of axes scores within a given treatment. Monte Carlo *P*-values for eigenvalues for the AM and EcM ordinations were 0.03 and <0.01, respectively. Joint-plot overlays were unable to detect any significant correlations between tree species and either the AM or EcM community composition.

CCA ordination of the AM TRFLP profiles explained 7.2% of the community variance, and the Monte Carlo *P*-value of 0.02 allows us to reject the null hypothesis of no relationship between the AM community and environmental factors. A separation between the regions but not treatments is evident in the ordination (Figure 1A) and these trends were confirmed with the MANOVA results (site *P*<0.01, treatment *P* = 0.22). Although 35 AM Terminal Restriction Fragment (TRF) peaks were detected, fragments that were 142 and 528 bp in length were present in nearly all samples and accounted for an average of 51% and 31% of the total peak area, respectively. The AM clone library yielded 89 sequences and BLAST results indicated that all AM sequences were from the genus *Glomus* although species could not be definitively determined. Grouping AM sequences by 97% sequence similarity revealed 25 different OTUs (Table S2). Thirteen of the 25 AM OTUs had predicted cut sites at 142 bp, matching with the most abundant TRF detected across the study; the second most abundant TRF at 528 bp was not predicted by any of the clones (Tables S2 and S3).

**Figure 2:**
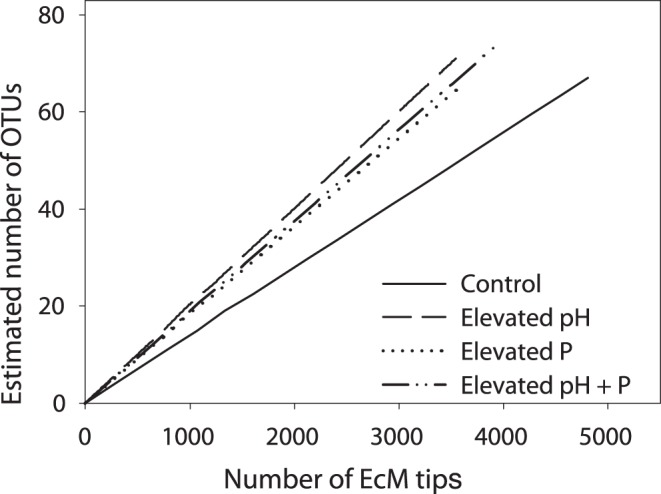
Rarefaction curves showing the expected number of species (97% OTUs) as a function of the number of EcM root tips from each treatment. Although the control appears to have lower diversity than the treatments, this visual difference is not statistically significant (95% confidence intervals not shown to improve figure clarity).

A total of 26,690 EcM root tips were counted and divided into 675 morphotypes. The number of root tips per plot varied greatly; six plots had no EcM tips (five of which were from the forest with 68% maple), while others had as many as 1,307 tips. The number of morphotypes was similarly variable with a maximum of 17 morphotypes in a given plot. Of the 675 morphotypes, all but 4 had successful amplification of the ITS region; however, gel electrophoresis revealed that 96 of the morphotypes had multiple ITS amplicons, leaving 575 samples that were submitted for sequencing. Of the samples submitted for sequencing, 193 were removed from the data set due to poor sequence quality (likely resulting from mixed template DNA), chimeric sequences, or sequences that were identified as non-EcM fungi. In all, we were able to successfully identify 382 (56%) of the morphotypes, representing 15,876 EcM tips, 26 genera and 219 OTUs (Table 2). The relative success rate of identifying morphotypes varied among treatments; elevated P plots had the lowest success rate at 50% while the control plots had the highest success rate at 67% (Table S4). Few sequences could be identified at the species level; however, most were identified to the genus level. Basidiomycetes accounted for 90% of the EcM tips with *Russula* and *Tomentella* being the most abundant genera accounting for 22% and 15% of the total root tips, respectively. CCA ordination of the EcM genera (Figure 1B) explained 10.4% of the community variance, and Monte Carlo tests indicated a significant relationship between EcM community and environmental factors (*P* = 0.05). The visual separation of regions and treatments was confirmed with the MANOVA results (site *P*<0.01, treatment *P*<0.01). Although there was a significant effect of region, EcM abundance and richness did not respond to treatments (Table 3). Of the 219 OTUs, 149 were found in only one of the 72 plots, thus not surprisingly the rarefaction curves did not plateau (Figure 2). Additionally, the rarefaction curves further confirm that while the treatments influenced the overall EcM community structure (Figure 1), they did not influence the number of expected OTUs.

**Table 2:** Successfully identified ectomycorrhizal taxa expressed as a percentage of the number of tips per treatment; total numbers of tips per treatment and per taxa are also presented.

	Treatment				Overall	
Taxa	Control	Elevated pH	Elevated P	Elevated pH+P	Total no. tips	Total percent
**Ascomycota**	**5.68**	**15.80**	**4.30**	**14.67**	**1566**	**9.86**
*Cenococcum*	0.0	0.0	0.20	4.23	174	1.10
*Genea*	0.0	0.45	0.0	0.08	19	0.12
*Helvella*	0.0	0.0	0.0	0.30	12	0.08
*Humaria*	0.81	0.0	0.0	0.0	39	0.25
*Lachnum*	1.58	0.0	0.0	0.0	76	0.48
*Pachyphloeus*	1.96	3.39	0.17	0.30	232	1.46
*Tuber*	0.0	3.67	0.67	3.37	287	1.81
Other & unknown	1.33	8.30	3.27	6.39	727	4.58
**Basidiomycota**	**94.32**	**84.20**	**95.70**	**85.33**	**14310**	**90.14**
*Amanita*	0.29	0.0	0.0	1.39	69	0.43
*Boletus*	0.12	0.0	0.17	0.56	34	0.21
*Clavulina*	1.08	0.48	7.04	9.55	698	4.40
*Cortinarius*	17.91	3.24	4.89	7.32	1440	9.07
*Hebeloma*	0.0	0.73	0.0	0.0	26	0.16
*Hydnum*	0.48	0.0	0.0	0.0	23	0.14
*Inocybe*	0.40	2.29	4.56	1.90	338	2.13
*Laccaria*	0.21	3.10	1.23	0.0	164	1.03
*Lactarius*	16.78	10.98	3.24	3.07	1433	9.03
*Piloderma*	0.85	0.25	9.31	0.0	383	2.41
*Russula*	22.90	21.56	26.44	17.38	3497	22.03
*Scleroderma*	0.40	0.0	0.25	0.0	28	0.18
*Sebacina*	4.01	6.12	5.76	11.94	1087	6.85
*Strobilomyces*	0.0	0.11	0.0	0.10	8	0.05
*Tomentella*	17.39	12.42	14.95	14.67	2390	15.05
*Tomentellopsis*	0.0	0.51	0.0	0.0	18	0.11
*Tricholoma*	0.0	0.0	0.0	7.05	278	1.75
Other & unknown	11.50	22.40	17.86	10.39	2396	15.09
**Total no. tips**	**4808**	**3544**	**3578**	**3946**	**15876**	

**Table 3:** The effect of region and treatments on tree roots and mycorrhizae with means and standard errors reported for each treatment.

Variable	*P* - value			Glaciated				Unglaciated			
	Region	Trt	Region x Trt	Control	Elevated pH	Elevated P	Elevated pH+P	Control	Elevated pH	Elevated P	Elevated pH+P
Woody roots(mg cm^−3^)	0.89	0.52	0.89	3.80 (0.14)	3.39(0.16)	3.14(0.15)	3.44(0.21)	3.26(0.17)	3.42(0.15)	3.00(0.10)	3.94(0.20)
AM root biomass(mg dry root cm^−3^)	0.82	0.12	0.95	0.18 (0.01)	0.28 (0.03)	0.40(0.04)	0.23(0.02)	0.18(0.02)	0.26(0.03)	0.34(0.02)	0.24(0.02)
AM colonization(gene copies mg^−1^ dry root)	0.77	0.06	0.66	1.77E5(1.98E4)	1.32E5(1.82E4)	8.03E4**(9.75E3)	7.32E4*(6.56E3)	1.54E5(2.47E4)	8.19E4(5.13E3)	4.73E4*(3.47E3)	9.10E5(7.78E3)
Total AM biomass (genecopies cm^−3^)	0.89	0.84	0.41	2.57E5 (2.45E4)	1.53E5(1.38E4)	1.97E5 (3.29E4)	1.49E5 (1.89E4)	1.50E5 (1.64E4)	1.29E5 (7.25E3)	1.63E5 (1.39E4)	1.74E5 (1.14E4)
EcM abundance(root tips cm^−3^)	0.02	0.50	0.98	0.22(0.03)	0.12(0.02)	0.12(0.01)	0.12(0.01)	0.63(0.04)	0.5(0.04)	0.51(0.06)	0.57(0.04)
EcM richness(OTUs plot^−1^)	0.10	0.88	0.74	3.11(0.24)	3.22(0.32)	3.00(0.26)	3.33(0.10)	5.78(0.15)	6.56(0.31)	5.22(0.21)	5.56(0.27)

*P*-values for the effect of region and treatments from the LME model with forest blocks as the random effect (n = 9). Asterisks denote a significant difference, in comparison to controls, at *P*<0.05 (**) and *P*<0.10 (*).

Total AM biomass calculated by multiplying AM root biomass by AM colonization.

There were no significant effects of region or treatment on the woody or AM root biomass (Table 3). Arbuscular mycorrhizal colonization (18S gene copies per g dry root) was significantly reduced by 40–45% in the glaciated elevated P and elevated pH+P treatments, and by ∼30% in the unglaciated elevated P treatment, compared to their respective controls. However, AM colonization per cm^3^ was not significantly different among treatments. EcM abundance and richness did not respond to treatments, although there was a significant effect of region (Table 3).

Although there was a significant treatment effect on soil pH and resin P (Table 1), not all plots responded equally to the treatments and several did not reach the target pH of 5.8 (i.e., Al^3+^ immobilization; data not shown). Therefore, Spearman Rank-Order correlations were used to examine the root and mycorrhizal response to pH and P availability (Table 4). Woody and AM roots had significant correlations only with the most available pool of P, resin P. Woody root biomass was negatively correlated with resin P (*P* = 0.05) and interestingly, while the biomass of AM roots was positively correlated to resin P (*P* = 0.01), AM colonization had a weak, negative correlation (*P* = 0.06). The most abundant AM TRF, 142, was negatively correlated with pH while the second most abundant TRF, 528, was negatively correlated with hydroxide P. On the other hand, EcM abundance and richness were negatively correlated with bicarb P and hydroxide P (*P*<0.01 for all). The relative abundance of EcM tips colonized by ascomycetes was positively correlated with pH (*P* = 0.05) and the relative abundance of EcM tips colonized by basidiomycetes was negatively correlated with hydroxide P (*P* = 0.02). Interestingly, the total AM colonization (18S gene copies cm^3^) was not significantly correlated with AM tree frequency (*P* = 0.44); although the number of EcM tips per cm^3^ was significantly correlated with frequency of EcM trees (correlation coefficient = 0.55, *P*<0.01).

**Table 4:** Root and mycorrhizal response to soil pH and P pools, shown as Spearman Rank-Order Correlation Coefficients.

	pH	Resin P	Bicarb P	Hydroxide P
Woody roots^a^	0.06	−0.23**	0.03	−0.04
AM root biomass^a^	0.13	0.31**	−0.01	0.12
AM colonization^a^	−0.03	−0.21*	0.05	0.02
AM TRF_142^b^	−0.27**	0.12	0.04	−0.11
AM TRF_528^b^	0.15	−0.13	0.14	0.26**
EcM abundance^a^	−0.01	−0.11	−0.34**	−0.59**
EcM richness^a^	0.08	−0.16	−0.31**	−0.53**
% Ascomycota EcM^c^	0.23**	−0.12	−0.03	0.05
% Basidiomycota EcM^c^	−0.18	0.18	−0.08	−0.27**

Asterisks denote significance at *P*<0.05 (**) and *P*<0.10 (*).

a Units as defined in Tables 1 and 3.

b Relative abundance of AM TRF peaks.

c Relative percent of Ascomycota and Basidiomycota EcM tips per plot.

## Discussion

Overall, one year after treatment initiation, the experimental treatments achieved the goal of increasing soil pH and P availability. Although not all of the lime amended plots reached the target pH of 5.8, all were greater than 5.0. Elevating P augmented the resin P pool, but had no effect on either the bicarb P or hydroxide P pools. When the TSP granules used in our treatments were analyzed with the P fractionation procedure, nearly all the P was recovered in the resin P pool (data not shown). Thus, it is not surprising that resin P is the only P pool impacted by our elevated P treatment.

Because EEAs are produced by soil microbes to decompose soil organic matter and liberate bound nutrients [57], the greater EEA in the glaciated region is likely a result of the higher microbial biomass in these soils [9]. Contrary to our hypothesis, EEAs did not respond to treatments. This is somewhat surprising given the findings of previous studies. Groffman and Fisk [3] found that amending forest soils with P decreased phosphatase activity in the O horizon. Additionally, using samples taken a few months prior to this study, DeForest et al. [9] reported significant treatment effects on the following enzymes: β-1,4-glucosidase; β-N-acetylglucosaminidase; leucine aminopeptidase; phosphomonoesterase; and phosphodiesterase. While it is not unusual to see seasonal variation in EEAs [54], [58], the difference between our findings and those of DeForest et al. [9] could also be due to differences in environmental conditions or sampling procedure. For example, unglaciated soil moisture was 22% for DeForest et al. [9], but decreased to 16% for this study (Table S1). The primary treatment effects reported in DeForest et al. [9] were in the unglaciated region, thus the lower soil mositure may have masked treatment effects due to abiotic suppression of microbial activity. In addition, DeForest et al. [9] sampled randomly within the plots, whereas we sampled 1 m from the base of trees where root biomass was greatest. This allowed us to focus on the treatment response of roots and mycorrhizae, but random sampling could have resulted in microbial activity estimates dominated more by saprotrophs than mycorrhizae. Taking samples in the more biologically active area near trees could also explain why we saw smaller resin P pools than previously reported. In DeForest et al. [9], resin P was 8.2 mg P kg^−1^ and 5.6 mg P kg^−1^, for glaciated and unglaciated elevated P plots, respectively, but 2.4 mg P kg^−1^ and 3.4 mg P kg^−1^ in our samples (Table 1). The elevated P treatment effects may have been weaker at the time we sampled because the readily available P was rapidly taken up by trees and rhizosphere organisms. However, because of the differences in sampling procedures, it is unclear whether the lack of an EEA response is due to temporal or rhizosphere effects.

Although our hypothesis that AM fungal communities would shift in response to treatments was not met, regional differences were detected. The relative dominance of *Acer* spp. in the glaciated forests ranges from 30–68%, but only 10–16% in unglaciated forests [9]; however, the difference in AM host dominance did not appear to influence the AM root biomass (Table 3). Because *Acer saccharum* and *Acer rubrum* are the dominant AM host trees in both regions, the greater number of AM hosts may have contributed to the regional differences in AM community structure. Additionally, the significant correlations between the two dominant AM TRFs with different soil properties (Table 4) further support that AM community structure is influenced by a combination of AM host abundance, spatial distribution, and soil factors. Indeed, Dumbrell et al. [31] found that dispersal limitation and soil factors have a combined influence on the composition of AM fungal communities. Soil pH has been shown to have a greater influence on AM fungi than plant host [31] and acidic soils reportedly have reduced diversity of AM fungi [30], [59]. The AM fungal communities presented here displayed low diversity using the TRFLP approach with two fragments accounting for over 31 and 51 percent of the total peak area across samples (Table S3). Although multiple taxa can produce fragments of the same size [60] our methods have previously been shown to be robust at detecting AM fungal community differences [38], and results from our clone library confirmed low AM diversity with only *Glomus* spp. represented. We hypothesize that while regional differences are present, soil acidity has reduced the taxonomic diversity of the AM fungi across the study area, and additional time may be necessary before a treatment effect becomes apparent.

Despite the lack of treatment effect on the AM community, we did observe a positive correlation between resin P and the biomass of AM roots that was accompanied by elevated P-induced declines in AM colonization. This suggests that when P is readily available, trees that form AM associations put more C towards increasing root biomass in lieu of supporting additional mycorrhizal symbionts. This finding mirrors those from an agricultural system where AM colonization decreased with increasing P availability [61]. Although AM colonization and root biomass did not respond to elevated pH in our study, previous studies have found varied responses. An 80 day seedling experiment by Van Aarle et al. [29] reported that AM colonization decreased with elevated pH, while a six-month seedling experiment reported that elevated pH increased AM colonization [30]. Additionally, a survey of healthy and declining *Acer saccharum* trees found that AM colonization was positively correlated with soil pH and that fine roots from declining trees had a lower P and Ca content compared to healthy trees [62]. However, Juice et al. [63] found that five years after the application of a calcium bearing mineral (wollastonite (CaSiO_3_)), AM root biomass and AM colonization increased, although soil pH was not dramatically altered, indicating that perhaps Ca nutrition might be a factor in determining AM colonization and root biomass responses when lime is used to elevate soil pH.

Morphotyping EcM root tips has long been used to assess EcM diversity; however, morphotyping is based on visual characteristics such as branching patterns, texure, and color, and thus several taxa may be inadvertently grouped into the same morphotype. To reduce the chances of this happening, we defined morphotypes within each plot, rather than across the entire study. Nevertheless, 96 of 675 morphotypes resulted in multiple PCR bands indicating a mixed template. Additionally, some sequence chromatograms appeared to have a mixed DNA template as well. Several studies have used cloning or repeated sequencing to assign the mixed morphotypes to multiple taxa [64], [65], while others have removed these mixed morphotypes from the analysis [21]. In this study, we chose to remove the mixed morphotypes from the analysis because it is impossible to determine the relative contribution of each member to the morphotype root tip count. Our identification of 56% of the EcM morphotypes is similar to the success rate reported by others [21], [64]. However, because our rarefaction curves did not reach an asymptote, increased sampling and/or sequencing efforts are needed to capture the full diversity of EcM fungi in these forests. It should also be noted that despite using similar methodology, the diversity of EcM reported in this study is much greater than reported by others examining the response of EcM fungi to N deposition and liming [21], [65]. This is likely because other studies examined EcM communities in stands with a single host species whereas our study was conducted in mixed forests with multiple EcM hosts (predominantly *Quercus*, *Fagus*, and *Betulacea*). Additionally, while some have found that soil pH has a greater effect on EcM communities than host composition [66], it has also been reported that within the *Quercus* genus, the host species can play a role in structuring EcM communities [33]. Our forests contain six different *Quercus* species with varying abundances in each region and forest, which may have further contributed to the high EcM richness reported here.

We found that EcM root tip abundance was negatively correlated with the two largest soil P pools, bicarb and hydroxide P. This would suggest that where soil P is more available, plants may be less dependent on EcM for P uptake. While regional differences in soil chemistry and EcM abundance likely impacted this result, correlation analysis on the glaciated region alone showed a similar trend (data not shown). Phosphorus availability has previously been linked to EcM community structure [32], [33], and while no specific taxa appeared to respond to the elevated P treatments, we did observe an overall shift in the EcM community, perhaps towards a community better adapted for an inorganic P economy. In addition, we saw significant negative correlations between EcM root tip abundance and species richness, suggesting that EcM colonization and richness may be greater in P-limited forests. EcM have been shown to have differing pH optima [67] and liming to increase soil pH and Ca has long been used as a management practice in truffle cultivation [68]; thus it is not surprising to see shifts in community composition in the elevated pH plots. The relative abundance of EcM tips colonized by Ascomycota was significantly correlated with soil pH, similar to the findings of Kjøller and Clemmensen [21] who found that sequences belonging to the Pezizales were more abundant in the limed plots of a boreal forest. Indeed, many of our Ascomycota sequences were also from the order Pezizales and included the truffle forming genera *Tuber* and *Pachyphloeus*. While our study occurred one year after treatment initiation, long-term effects of forest liming on EcM have been reported. Rineau et al. [66] found altered EcM communities 15 years after lime application and Kjøller and Clemmensen [21] reported shifts in EcM community composition 16 years after lime addition. We expect that the EcM fungal communities will continue to adjust to the new environmental conditions created by the treatments and continued monitoring is planned.

In summary, within one year of implementing an ecosystem-level experiment to alter soil pH and P availability in temperate hardwood forests, we observed significant changes in EcM fungal communities, AM colonization, and root growth. Our findings also indicate that AM and EcM fungi responded to different soil P pools: AM roots and colonization respond to resin P pools and EcM fungi respond to the bicarb and hydroxide P pools. Although all P pools measured are considered biologically available, more metabolic effort is required for microbes to obtain the bicarb and hydroxide pools compared to the resin pool. Furthermore, mycorrhizae vary in their ability to acquire P from different sources and our results suggest that P availability and source have a combined influence on mycorrhizal fungi. The overall results of our hypothesis testing are mixed: while the EcM community responded to treatment, the abundance of EcM root tips did not. In contrast, the AM community did not respond to treatments but AM colonization did. Our findings highlight the complex interactions between mycorrhizae, their hosts, and the soil environment and further underscore the need to better understand the influence of ecosystem acidification and P availability on temperate forest mycorrhizae. Additional work is needed to explore how microbial communities and activities vary seasonally within these forest treatments.

## Supporting Information

Table S1 **The effect of region and treatments on soil properties with means and standard errors reported for each treatment.**
(DOCX)

Table S2 **Distribution of OTUs from AM clone library; predicted** ***Hinf*** I cut site from in silico digest of consensus sequence, best BLAST hit*, and accession numbers also shown.
(DOCX)

Table S3 **Average AM TRF abundance (percent of total peak area per plot) for the most abundant HinfI TRF fragments.** Rare fragments that occurred in less than five plots are not included in this table although they are included in the community analysis.
(DOCX)

Table S4 **Success of EcM identification across treatments.**
(DOCX)
