# Supplementary material for: Mycorrhizal Response to Experimental pH and P Manipulation in Acidic Hardwood Forests
Source: PLoS One. 2012 Nov 8;7(11):e48946. doi: 10.1371/journal.pone.0048946 (PMC3493595; doi:10.1371/journal.pone.0048946)
Supplement: Table S1 — The effect of region and treatments on soil properties with means and standard errors reported for each treatment. (DOCX) [file pone.0048946.s001.docx]

**Table S1.** The effect of region and treatments on soil properties with means and standard errors reported for each treatment.

| Variable | *P* - value | | | Glaciated | | | | Unglaciated | | | |
| --- | --- | --- | --- | --- | --- | --- | --- | --- | --- | --- | --- |
|  | Region | Trt | Region x Trt | Control | Elevated pH | Elevated P | Elevated pH + P | Control | Elevated pH | Elevated P | Elevated pH + P |
| Water content  (%) | 0.06 | 0.90 | 0.67 | 28.6  (0.9) | 27.9  (1.0) | 29.3 (1.1) | 29.1  (1.3) | 15.9  (0.7) | 16.6  (0.5) | 17.2  (0.5) | 15.6  (0.6) |
| Total N  (mg N kg^-1^) | 0.03 | 0.69 | 0.63 | 4.27  (0.16) | 3.84  (0.14) | 4.12  (0.17) | 3.94  (0.21) | 2.22  (0.04) | 2.44  (0.07) | 2.63*  (0.07) | 2.38  (0.05) |
| Total C  (mg C kg^-1^) | 0.04 | 0.92 | 0.39 | 71.20 (2.90) | 62.72  (2.12) | 54.15  (2.47) | 64.54  (3.31) | 38.65  (0.66) | 39.64  (0.52) | 42.82  (0.63) | 43.61  (0.50) |
| Molar C:N | 0.25 | 0.59 | 0.88 | 22.18  (0.27) | 21.98  (0.23) | 20.87  (0.28) | 21.94  (0.24) | 23.79  (0.54) | 22.59  (0.57) | 22.73  (0.62) | 25.12  (0.51) |
| β-1,4-Glucosidase | <0.01 | 0.57 | 0.99 | 355.73  (28.31) | 427.61  (34.90) | 456.08  (41.36) | 414.54  (31.92) | 118.11  (8.99) | 103.97  (5.65) | 126.09  (8.02) | 113.98  (11.39) |
| Cellobiohydrolase | <0.01 | 0.23 | 0.94 | 79.15  (6.84) | 96.93  (7.13) | 104.30  (9.70) | 90.11  (7.84) | 17.98  (2.61) | 13.64  (1.00) | 17.42  (1.68) | 10.43  (1.12) |
| β-N-acetylglucosaminidase | 0.03 | 0.99 | 0.80 | 601.72  (77.03) | 430.23  (52.05) | 418.13  (25.62) | 368.92  (34.13) | 226.02  (17.79) | 145.81  (5.44) | 177.72  (9.22) | 220.4  (10.04) |
| Leucine aminopeptidase | <0.01 | 0.48 | 0.61 | 63.77  (8.84) | 85.84  (15.60) | 38.61  (1.21) | 43.93  (1.67) | 15.87  (0.31) | 15.30  (0.32) | 18.32  (0.62) | 18.52  (1.25) |
| Phosphomonoesterase | <0.01 | 0.19 | 0.96 | 1778.90  (88.93) | 1517.40  (76.51) | 1722.05  (79.48) | 1592.11  (71.15) | 552.97  (16.20) | 441.34  (8.63) | 508.23  (17.25) | 491.39  (15.10) |
| Phosphodiesterase | <0.01 | 0.04 | 0.96 | 184.40  (9.43) | 145.60  (8.24) | 160.32  (7.76) | 137.14  (7.22) | 49.22  (2.05) | 36.05  (0.79) | 38.35  (1.31) | 37.07  (1.32) |

EEA activities reported as nmol g soil^-1^ hr^-1^

*P* - values for the effect of region and treatments from the LME model with forest blocks as the random effect (n = 9).

Asterisks denote a significant difference, in comparison to controls, at *P* < 0.05 (**) and *P* < 0.10 (*).
