# Supplementary material for: Mycorrhizal Response to Experimental pH and P Manipulation in Acidic Hardwood Forests
Source: PLoS One. 2012 Nov 8;7(11):e48946. doi: 10.1371/journal.pone.0048946 (PMC3493595; doi:10.1371/journal.pone.0048946)
Supplement: Table S2 — Distribution of OTUs from AM clone library; predicted Hinf I cut site from in silico digest of consensus sequence, best BLAST hit*, and accession numbers also shown. (DOCX) [file pone.0048946.s002.docx]

**Table S2.** Distribution of OTUs from AM clone library; predicted *Hinf*I cut site from in silico digest of consensus sequence, best BLAST hit*, and accession numbers also shown

| AM OTU | Pairwise Identity of OTU (%) | Number of Clones in Library | Predicted *Hinf*I cut site (bp) | Best BLAST hit  (name, accession number, % similarity to query sequence) | Accession Numbers |
| --- | --- | --- | --- | --- | --- |
| OTU 1 | 98.9 | 2 | 259 | Glomeromycota sp. MIB 8446, JF414180.1, 96% | JQ654497 - JQ654498 |
| OTU 2 | 99.8 | 2 | 117 | *Glomus coronatum*, FR773145.1, 96% | JQ654499 - JQ654500 |
| OTU 3 | 99.4 | 4 | 191 | Glomeromycota sp. MIB 8376, JF414177.1, 97% | JQ654501 - JQ654504 |
| OTU 4 | 99.1 | 3 | 190 | Glomeromycota sp. MIB 8376, JF414177.1, 98% | JQ654505 - JQ654507 |
| OTU 5 | 99.6 | 6 | 525 | *Glomus macrocarpum*, FR750376.1, 98% | JQ654508 - JQ654513 |
| OTU 6 | 98.8 | 3 | 142 | *Glomus* sp. 8451.1, JQ811204.1, 98% | JQ654514 - JQ654516 |
| OTU 7 | 98.8 | 4 | 142 | *Rhizophagus cf. irregularis*, FR750223.1, 99% | JQ654517 - JQ654520 |
| OTU 8 | 100 | 2 | 142 | Glomeromycota sp. MIB 8370, JF414193.1, 97% | JQ654521 - JQ654522 |
| OTU 9 | 99.9 | 5 | 142 | *Glomus clarum*, FR773148.1, 97% | JQ654523 - JQ654527 |
| OTU 10 | 100 | 9 | 142 | Glomeromycota sp. MIB 8446, JF414180.1, 97% | JQ654528 - JQ654536 |
| OTU 11 | 99.5 | 7 | 142 | Glomeromycota sp. MIB 8366, JF414187.1, 96% | JQ654537 - JQ654543 |
| OTU 12 | 99.6 | 10 | 142 | Glomeromycota sp. MIB 8446, JF414180.1, 96% | JQ654544 - JQ654553 |
| OTU 13 | 100 | 2 | 142 | *Glomus* sp. 8451.1, JQ811204.1, 97% | JQ654554 - JQ654555 |
| OTU 14 | 98.8 | 19 | 142 | *Glomus* sp. ST10, JQ811203.1, 97% | JQ654556 - JQ654574 |
| OTU 15 | - | 1 | 281 | *Glomus clarum*, FR773148.1, 96% | JQ654575 |
| OTU 16 | - | 1 | 190 | *Glomus macrocarpum*, FR772325.1, 95% | JQ654576 |
| OTU 17 | - | 1 | 142 | Glomeromycota sp. MIB 8376, JF414177.1, 96% | JQ654577 |
| OTU 18 | - | 1 | 191 | Glomeromycota sp. MIB 8370, JF414193.1, 96% | JQ654578 |
| OTU 19 | - | 1 | 142 | *Rhizophagus cf. irregularis*, FR750223.1, 97% | JQ654579 |
| OTU 20 | - | 1 | 525 | Glomeromycota sp. MIB 8366, JF414187.1, 97% | JQ654580 |
| OTU 21 | - | 1 | 190 | *Glomus* sp. MS Appelhans, EU518489.1, 98% | JQ654581 |
| OTU 22 | - | 1 | 142 | *Glomus* sp. 8451.1, JQ811204.1, 98% | JQ654582 |
| OTU 23 | - | 1 | 142 | Glomeromycota sp. MIB 8446, JF414180.1, 96% | JQ654583 |
| OTU 24 | - | 1 | 132 | *Glomus clarum*, FR773148.1, 95% | JQ654584 |
| OTU 25 | - | 1 | 141 | Glomeromycota sp. MIB 8369, JF414192.1, 97% | JQ654585 |

*To avoid poorly identified or mis-named sequences, best BLAST hits were identified using the NCBI database with the Entrez query filter “src specimen voucher[properties]” to compare sequences to voucher specimens only.
