# Supplementary material for: Mycorrhizal Response to Experimental pH and P Manipulation in Acidic Hardwood Forests
Source: PLoS One. 2012 Nov 8;7(11):e48946. doi: 10.1371/journal.pone.0048946 (PMC3493595; doi:10.1371/journal.pone.0048946)
Supplement: Table S3 — Average AM TRF abundance (percent of total peak area per plot) for the most abundant HinfI TRF fragments. Rare fragments that occurred in less than five plots are not included in this table although they are included in the community analysis. (DOCX) [file pone.0048946.s003.docx]

**Table S3**. Average AM TRF abundance (percent of total peak area per plot) for the most abundant HinfI TRF fragments. Rare fragments that occurred in less than five plots are not included in this table although they are included in the community analysis.

|  | Glaciated | | | | Unglaciated | | | |  |
| --- | --- | --- | --- | --- | --- | --- | --- | --- | --- |
| Peak | Control | Elevated P | Elevated pH | Elevated pH+P | Control | Elevated P | Elevated pH | Elevated pH+P | Overall percent |
| TRF_142 | 49.24 | 57.13 | 47.87 | 61.97 | 47.99 | 47.82 | 42.19 | 57.11 | 51.25 |
| TRF_528 | 40.43 | 30.69 | 36.15 | 26.31 | 27.62 | 30.27 | 33.12 | 25.71 | 31.32 |
| TRF_527 | 18.03 | 9.401 | 0 | 0 | 21.09 | 17.84 | 7.93 | 25.12 | 17.01 |
| TRF_191 | 13.94 | 8.9 | 6.50 | 3.97 | 10.78 | 10.9 | 9.44 | 6.71 | 8.8 |
| TRF_192 | 3.02 | 3.38 | 5.50 | 4.36 | 4.52 | 6.12 | 12.16 | 6.45 | 6.01 |
| TRF_282 | 0 | 0 | 9.62 | 0 | 4.15 | 0 | 4.713 | 0 | 5.47 |
| TRF_159 | 2.68 | 3.04 | 2.41 | 7.17 | 8.07 | 0.0 | 0 | 1.6 | 3.59 |
| TRF_281 | 2.46 | 1.35 | 1.56 | 0 | 2.93 | 4.25 | 2.27 | 6.34 | 3.32 |
| TRF_132 | 1.84 | 3.25 | 1.45 | 5.55 | 0 | 0 | 0 | 0 | 2.79 |
| TRF_56 | 0 | 2.14 | 1.78 | 1.38 | 2.415 | 2.93 | 2.46 | 2.27 | 2.38 |
| TRF_138 | 1.94 | 1.92 | 1.79 | 2.13 | 2.95 | 2.52 | 2.48 | 2.55 | 2.33 |
| TRF_52 | 0 | 1.39 | 1.29 | 1.35 | 2.29 | 2.78 | 2.25 | 1.94 | 2.17 |
| TRF_137 | 1.16 | 0 | 2.74 | 1.53 | 2.07 | 0 | 0 | 0 | 1.76 |
| TRF_525 | 0 | 1.56 | 1.17 | 1.01 | 3.15 | 0.0 | 1.01 | 1.93 | 1.57 |
| TRF_188 | 0 | 0 | 1.24 | 0 | 1.02 | 1.13 | 0 | 1.41 | 1.24 |
