# Supplementary material for: Mycorrhizal Response to Experimental pH and P Manipulation in Acidic Hardwood Forests
Source: PLoS One. 2012 Nov 8;7(11):e48946. doi: 10.1371/journal.pone.0048946 (PMC3493595; doi:10.1371/journal.pone.0048946)
Supplement: Table S4 — Success of EcM identification across treatments. (DOCX) [file pone.0048946.s004.docx]

**Table S4.** Success of EcM identification across treatments.

|  | Number of EcM root tips | | | | | Morphotypes |
| --- | --- | --- | --- | --- | --- | --- |
|  | Control | Elevated pH | Elevated P | Elevated pH+P | Total |  |
| Starting | 7128 | 5896 | 7056 | 6610 | 26690 | 675 |
| No amplification | 21 | 20 | 0 | 11 | 52 | 4 |
| Double bands | 997 | 903 | 1612 | 515 | 4027 | 96 |
| Poor sequence | 1302 | 1419 | 1866 | 2138 | 6735 | 193 |
|  |  |  |  |  |  |  |
| Total successfully identified | 4808 | 3554 | 3578 | 3946 | 15876 | 382 |
| % Successfully identified | 67.45 | 60.28 | 50.71 | 59.69 | 59.48 | 56.59 |
